# Clinical and radiographic predictors of successful therapeutic bronchoscopy for the relief of malignant central airway obstruction

**DOI:** 10.1186/s12890-019-0987-3

**Published:** 2019-11-21

**Authors:** Coral X. Giovacchini, Edward R. Kessler, Christopher M. Merrick, Junheng Gao, Xiaofei Wang, Momen M. Wahidi, Scott L. Shofer, George Z. Cheng, Kamran Mahmood

**Affiliations:** 1 Division of Pulmonary, Allergy and Critical Care Medicine, Department of Medicine, Duke University Medical Center, Durham, NC USA; 2 Interventional Pulmonology, Division of Pulmonary & Critical Care Medicine, Duke University Health System, Duke Cancer Center Raleigh, 3404 Wake Forest Road, Suite 303, Raleigh, NC 27609 USA; 3 Interventional Pulmonary Medicine, Chicago Chest Center, Suburban Lung Associates, Elk Grove Village, IL USA; 4 Division of Pulmonary and Critical Care Medicine, Department of Medicine, Vanderbilt University, Nashville, TN USA; 5 Department of Statistics, Duke University Medical Center, Durham, NC USA

**Keywords:** Airway obstruction, Bronchial neoplasms, Bronchoscopy, Pulmonary surgical procedures, Tracheal stenosis

## Abstract

**Background:**

Malignant central airway obstruction (CAO) occurs in approximately 20–30% of patients with lung cancer and is associated with debilitating symptoms and poor prognosis. Multimodality therapeutic bronchoscopy can relieve malignant CAO, though carries risk. Evidence to guide clinicians regarding which patients may benefit from such interventions is sparse. We aimed to assess the clinical and radiographic predictors associated with therapeutic bronchoscopy success in relieving malignant CAO.

**Methods:**

We reviewed all cases of therapeutic bronchoscopy performed for malignant CAO at our institution from January 2010–February 2017. Therapeutic bronchoscopy success was defined as establishing airway patency of > 50%. Patient demographics and baseline characteristics, oncology history, degree of airway obstruction, procedural interventions, and complications were compared between successful and unsuccessful groups. Univariate and multivariate logistic regression identified the significant clinical and radiographic predictors for therapeutic success. The corresponding simple and conditional odds ratio were calculated. A time-to-event analysis with Kaplan–Meier plots was performed to estimate overall survival.

**Results:**

During the study period, 301 therapeutic bronchoscopies were performed; 44 (14.6%) were considered unsuccessful. Factors associated with success included never vs current smoking status (OR 5.36, 95% CI:1.45–19.74, *p* = 0.010), patent distal airway on CT imaging (OR 15.11, 95% CI:2.98–45.83, *p* < 0.0001) and patent distal airway visualized during bronchoscopy (OR 10.77, 95% CI:3.63–31.95, *p* < 0.001) in univariate analysis. Along with patent distal airway on CT imaging, increased time from radiographic finding to therapeutic bronchoscopy was associated with lower odds of success in multivariate analysis (OR 0.96, 95% CI:0.92–1.00, *p* = 0.048). Median survival was longer in the successful group (10.2 months, 95% CI:4.8–20.2) compared to the unsuccessful group (6.1 months, 95% CI:2.1–10.8, log rank *p* = 0.015).

**Conclusions:**

Predictors associated with successful therapeutic bronchoscopy for malignant CAO include distal patent airway visualized on CT scan and during bronchoscopy. Odds of success are higher in non-smokers, and with decreased time from radiographic finding of CAO to intervention.

## Introduction

Central airway obstruction (CAO) can result from a variety of malignant and non-malignant disorders [1] and is generally defined as > 50% obstruction of the trachea, mainstem bronchi, bronchus intermedius or lobar bronchi [1–4]. Malignant CAO occurs more frequently than non-malignant obstruction [5], and is estimated to occur in approximately 20–30% of patients with primary lung cancer, often presenting as late stage or recurrent loco-regional disease [1, 6–10]. In addition, malignant CAO can occur in patients with metastatic disease from non-pulmonary malignancies, including breast, colon, thyroid, and renal cancers, among others [1, 4]. In all cases, malignant CAO is considered a serious and life-threatening complication, often resulting in dyspnea, hemoptysis, atelectasis, obstructive pneumonia, and respiratory failure, leading to decreased functional status, reduced quality of life and increased mortality [8, 9, 11, 12].

Therapeutic bronchoscopy with tumor debridement, ablative therapies, stent placement, and other modalities has been shown to palliate symptoms, improve spirometry, functional capacity, quality of life and survival in patients with malignant CAO [5, 6, 8–11, 13–17]. However, therapeutic bronchoscopy is a high-risk procedure, often performed under general anesthesia and with complications including bleeding, hypoxia, airway perforation, respiratory failure and death [2, 6, 18–20]. Further, many patients presenting with malignant CAO may have significantly impaired functional status, particularly respiratory reserve, when considering therapeutic bronchoscopy [13, 18, 19]. Better defining which patients may benefit from such interventions is paramount; however, evidence to guide clinicians is sparse. We aimed to assess the clinical and radiographic predictors associated with therapeutic bronchoscopy success in relieving malignant CAO.

## Methods

We retrospectively reviewed all cases of therapeutic bronchoscopy performed for symptomatic malignant CAO at our single institution between January 2010 and February 2017. Malignant CAO was defined as a luminal obstruction of > 50% in the trachea, mainstem bronchi, bronchus intermedius and/or a lobar bronchus, consistent with previous major studies [13, 18]. All therapeutic interventions were performed at the discretion of the attending bronchoscopist. Therapeutic success was defined as the ability to establish airway lumen patency > 50% during the bronchoscopic intervention [13, 18]. All unsuccessful cases were included; comparative successful cases were randomly selected from the data set for analysis. Since the randomly selected patients in the successful group did not have any significant difference from the unsuccessful group in age, gender, race, BMI, type of malignancy and comorbid lung diseases including COPD, asthma and interstitial lung disease, propensity score matching was not performed to avoid introducing any potential bias. This study was approved by the Duke University Institutional Review Board (Pro00100722).

### Therapeutic bronchoscopy procedure

Therapeutic bronchoscopy at our institution was performed in the operating room, and a rigid bronchoscope (Bryan Corporation, Woburn, Mass., USA) was inserted into the airway via the mouth under general anesthesia. All patients were ventilated using an automated jet ventilator (Bear Jet-150). The rigid telescope, flexible bronchoscope and other instruments utilized during the procedure were inserted via the primary lumen of the rigid bronchoscope for therapeutic intervention, as we have previously described [3]. In general, intrinsic or endobronchial lesions were debrided using multiple modalities. Argon plasma coagulation (APC) and rigid or flexible electrocautery (ERBE USA, Inc., Marietta GA, USA) were preferred heat modalities utilized for tumor ablation and/or hemostasis during tumor debridement. Stent placement was performed, when necessary, for extrinsic or mixed compressive lesions, with metal (Ultraflex Boston Scientific, Marlborough, MA, USA), hybrid (AERO, Merit Medical Endotek, South Jordan, UT, USA) or silicone (NOVATECH Dumon, Boston Medical Products, Inc., Westborough, MA, USA) stents chosen at the discretion of the bronchoscopist based on the type and location of the airway obstruction [3]. The average duration of the therapeutic rigid bronchoscopy procedure at our institution was 60 min.

### Data collected

Information including basic demographics, smoking, pulmonary comorbidities, malignant tumor histology and oncologic treatment history was extracted from patient charts. Bronchoscopic airway obstruction data including severity of obstruction (%lumen occluded), endo-luminal location, type of obstruction (extrinsic, intrinsic or mixed), patency of visualized airways distal to the obstruction, technical aspects of the procedure, and degree of therapeutic success after the intervention was extracted from the documented procedure notes. Radiographic studies prior to and after the intervention were reviewed for airway patency and radiographic improvement by independent reviewers not previously involved in the procedures. Patient charts were reviewed for any procedure-related complications including hypoxic respiratory failure, pneumomediastinum, pneumothorax, bleeding, infection, stent migration or death occurring as a direct result of the procedure within 72 h. Overall survival was calculated as the time from therapeutic bronchoscopy to death or date of last patient follow up.

### Statistical analysis

Univariate analyses, including chi-square, Mantel Haenszel tests and Kruskal-Wallis tests were performed to test the association between therapeutic success of bronchoscopy and the above defined unordered categorical, ordered categorical and continuous explanatory variables, respectively. Multivariate logistic regression was built with clinically relevant and statistically significant predictors of therapeutic success from simple odds ratio calculation. Median survival and associated 95% confidence intervals were estimated with Kaplan-Meier method. Log-rank testing was used to evaluate survival differences for therapeutic success and failure groups. Statistical analyses were conducted using SAS version 9.4 (Cary, NC).

## Results

From January 2010 through February 2017, 301 therapeutic bronchoscopies were performed at our institution for malignant CAO; 44 (14.6%) procedures were unsuccessful in establishing airway lumen patency. Unsuccessful cases were compared with 50 randomly selected successful cases (total *n* = 94). Baseline patient characteristics are presented in Table 1. Between the successful and unsuccessful groups, there was no difference in patient characteristics including patient age, sex, race, type of malignancy, underlying lung disease or previous therapies used for treatment of underlying malignancy. Smoking status was associated with a significant difference between successful and unsuccessful therapeutic bronchoscopy groups (*p* = 0.032). Compared to current smokers, never smokers had higher odds of successful therapeutic bronchoscopy (OR 5.36, 95% CI: 1.45–19.74, *p* = 0.010). There was a non-significant statistical trend toward higher odds of success in former smokers compared to current smokers (OR 2.61, 95% CI: 0.91–7.48, *p* = 0.072). Importantly, odds of successful therapeutic bronchoscopy were not significantly different between never smokers and former smokers (OR 2.05, 95% CI: 0.69–6.14, *p* = 0.197). Although there were less current and former smokers in the successful vs. unsuccessful group (70% vs. 86%, *p* = 0.03), the prevalence of COPD was similar in both groups (40% vs. 36%, *p* = 0.71).

**Table 1:** Patient Demographics

Characteristic	All (*n* = 94)	Successful Therapeutic Bronchoscopy (*n* = 50)	Unsuccessful Therapeutic Bronchoscopy (*n* = 44)	*p* value
Age – yr	60.6 + 12.4	59.7 + 14.2	61.5 + 10.1	0.955
Male Sex - no. (%)	55 (58.5)	29 (58.0)	26 (59.1)	0.915
Body Mass Index - kg/m2	27.0 + 7.6	27.4 + 7.8	26.7 + 7.6	0.682
Race				0.200
Caucasian	72 (76.6)	37 (74.0)	35 (79.5)	
African American	14 (14.9)	6 (12.0)	8 (18.2)	
Indian	2 (2.1)	2 (4.0)	–	
Unknown	6 (6.4)	5 (10.0)	1 (2.3)	
Smoking Status				0.032*
Current	22 (23.4)	7 (14.0)	15 (34.1)	
Former	51 (54.3)	28 (56.0)	23 (52.3)	
Never	21 (22.3)	15 (30.0)	6 (13.6)	
Underlying Lung Disease				0.637
None	52 (55.3)	26 (52.0)	26 (59.1)	
COPD	36 (38.3)	20 (40.0)	16 (36.4)	
IPF	1 (1.1)	1 (2.0)	–	
NSIP	1 (1.1)	1 (2.0)	–	
Asthma	3 (3.2)	2 (4.0)	1 (2.3)	
Mesothelioma	1 (1.1)	–	1 (2.3)	
Peri-procedural Length of Hospitalization - days	5.0 + 7.9	3.3 + 4.9	4.1 + 6.5	0.324
Current Chemotherapy				0.081
Yes	13 (13.8)	4 (8.0)	9 (20.5)	
No	81 (86.2)	46 (92.0)	35 (79.5)	
Current Radiation Therapy				0.346
Yes	1 (1.1)	1 (2.0)	–	
No	93 (98.9)	49 (98.0)	44 (100)	
Previous Chemotherapy				0.428
Yes	47 (50.0)	22 (44.0)	25 (56.8)	
No	43 (45.7)	26 (52.0)	17 (38.6)	
Unknown	4 (4.3)	2 (4.0)	2 (4.5)	
Previous Radiation Therapy				0.358
Yes	16 (17.0)	6 (12.0)	10 (22.7)	
No	70 (74.5)	40 (80.0)	30 (68.2)	
Unknown	8 (8.5)	4 (8.0)	4 (9.1)	
Adjuvant Chemotherapy				0.710
Yes	43 (45.7)	21 (42.0)	22 (50.0)	
No	38 (40.4)	22 (44.0)	16 (36.3)	
Unknown	13 (13.8)	7 (14.0)	6 (13.6)	
Adjuvant Radiation Therapy				0.400
Yes	41 (43.6)	19 (38.0)	22 (50.0)	
No	41 (43.6)	25 (50.0)	16 (36.3)	
Unknown	12 (12.7)	6 (12.0)	6 (13.6)	
Peri-procedural Antibiotics				0.770
Yes	16 (17.0)	8 (16.0)	8 (18.0)	
No	78 (82.9)	42 (84.0)	36 (81.8)	

Data shown as mean + SD; *p < 0.05 compared to unsuccessful therapeutic bronchoscopy group

The tumor characteristics found during therapeutic bronchoscopy are summarized in Table 2. There was no significant difference in tumor origin (lung vs. metastatic), tumor histology, non-small cell lung cancer (NSCLC) pathology, location of the obstruction, or type of obstruction (extrinsic, intrinsic or mixed) between successful and unsuccessful groups. However, radiographic distal airway patency, defined as patent airway visualized distal to the central airway obstruction site on chest CT, with or without lung atelectasis, was more common in the successful group (66% vs. 11.4%, *p* < 0.001) and associated with significantly high odds of success (OR 15.11, 95% CI: 2.98–45.83, *p* < 0.0001).

**Table 2:** Tumor Characteristics

Characteristic	All (*n* = 94)	Successful Therapeutic Bronchoscopy (*n* = 50)	Unsuccessful Therapeutic Bronchoscopy (*n* = 44)	*p* value
Tumor Origin				0.771
Lung	67 (71.3)	35 (70.0)	32 (72.7)	
Metastatic (from other areas)	27 (28.7)	15 (30.0)	12 (27.3)	
Tumor Histology				0.203
NSCLC	54 (57.4)	26 (52.0)	28 (63.6)	
SCLC	6 (6.4)	2 (4.0)	4 (9.1)	
Breast	2 (2.1)	–	2 (4.5)	
Carcinoid	7 (7.4)	7 (14.0)	–	
Gastrointestinal	6 (6.4)	3 (6.0)	3 (6.8)	
Lymphoma	1 (1.1)	1 (2.0)	–	
Melanoma	1 (1.1)	1 (2.0)	–	
Mesothelioma	1 (1.1)	–	1 (2.3)	
Pheochromocytoma	1 (1.1)	–	1 (2.3)	
Renal	9 (9.6)	6 (12.0)	3 (6.8)	
Sarcoma	3 (3.2)	2 (4.0)	1 (2.3)	
Thymoma	1 (1.1)	1 (2.0)	–	
Uterine	2 (2.1)	1 (2.0)	1 (2.3)	
NSCLC Pathology				0.232
Adenocarcinoma	15 (27.8)	6 (23.1)	9 (32.1)	
Large Cell	4 (7.4)	1 (3.8)	3 (10.7)	
Poorly Differentiated	2 (3.7)	–	2 (7.1)	
Squamous Cell	33 (61.1)	19 (73.1)	14 (50.0)	
Obstruction Type				0.600
Extrinsic	10 (10.6)	6 (12.0)	4 (9.1)	
Intrinsic	68 (72.3)	34 (68.0)	34 (77.3)	
Mixed	16 (17.0)	10 (20.0)	6 (13.6)	
Mass Location (specific)				0.068
Bronchus Intermedius	14 (14.9)	6 (12.0)	8 (18.2)	
Left Mainstem	30 (31.9)	15 (30.0)	15 (34.1)	
Lobar	18 (19.1)	6 (12.0)	12 (27.3)	
Right Mainstem	25 (26.6)	17 (34.0)	8 (18.2)	
Trachea	7 (7.4)	6 (12.0)	1 (2.3)	
Mass Location (general)				0.060
Lobar	18 (19.1)	6 (12.0)	12 (27.3)	
Non-Lobar	76 (80.9)	44 (88.0)	32 (72.7)	
Radiographic Distal Airway Patency				< 0.001*
Yes	36 (39.6)	31 (66.0)	5 (11.4)	
No	55 (60.4)	16 (34.0)	39 (88.6)	
Unknown	3 (−)	3 (−)	–	

Data shown as number (%); *p < 0.05 compared to unsuccessful therapeutic bronchoscopy group

The procedural findings and modalities used during therapeutic bronchoscopy are summarized in Table 3. Time from radiographic diagnosis to intervention was 14.7 days in the successful group vs. 17.2 days in the unsuccessful group, *p* = 0.71. Percentage of luminal airway obstruction as assessed on bronchoscopy was less in the successful group compared to the unsuccessful group (90% + 10% vs. 100% + 10%, *p* < 0.001). Odds of therapeutic success significantly decreased with each 10% increase in severity of obstruction (OR 0.36, 95% CI: 0.34–0.39, *p* < 0.0001). Distal airway patency on bronchoscopy was seen in 58% patients in the successful group vs. 11.4% patients in the unsuccessful group, *p* < 0.001. Visualizing a distal patent airway beyond the CAO during bronchoscopy was associated with significantly increased odds of therapeutic success (OR 10.77, 95% CI: 3.63–31.95, *p* < 0.001). Multiple therapeutic modalities were used in the majority of bronchoscopies, including a combination of debridement and ablative therapies, including APC, electrocautery and potassium titanyl phosphate (KTP) laser.

**Table 3:** Procedural Aspects & Techniques

Characteristic	All (*n* = 94)	Successful Therapeutic Bronchoscopy (*n* = 50)	Unsuccessful Therapeutic Bronchoscopy (*n* = 44)	*p* value
Time to Intervention (days) From:				
Radiographic Finding	15.9 + 16.0	14.7 + 14.5	17.2 + 17.7	0.711
Symptom Onset	69.2 + 86.2	60.1 + 68.9	79.2 + 101.8	0.942
Severity of Obstruction (%)	90.0 + 10.0	90.0 + 10.0	100 + 10.0	< 0.001*
Bronchoscopic Distal Airway Patency				< 0.001*
Ys	34 (36.2)	29 (58.0)	5 (11.4)	
No	60 (63.8)	21 (42.0)	39 (88.6)	
Mechanical Debridement				0.763
Yes	76 (80.9)	41 (82.0)	35 (79.5)	
No	18 (19.1)	9 (18.0)	9 (20.5)	
Argon Plasma Coagulation				0.016*
Yes	39 (41.5)	15 (30.0)	24 (54.4)	
No	55 (58.5)	35 (70.0)	20 (45.5)	
Cryodebridement				0.484
Yes	3 (3.2)	1 (2.0)	2 (4.5)	
No	91 (96.8)	49 (98.0)	42 (95.5)	
Electrocautery				0.908
Yes	54 (57.4)	29 (58.0)	25 (56.8)	
No	40 (42.6)	21 (42.0)	19 (43.2)	
KTP Laser Therapy				0.004*
Yes	12 (12.8)	11 (22.0)	1 (2.3)	
No	82 (87.2)	39 (78.0)	43 (97.7)	
Stent Placement				< 0.001*
Yes	27 (28.7)	27 (54.0)	–	
No	67 (71.3)	23 (46.0)	44 (100)	
Radiographic Improvement				< 0.001*
Yes	53 (56.4)	39 (78.0)	14 (31.8)	
No	41 (43.6)	11 (22.0)	30 (68.2)	
Complications				0.466
None	87 (92.6)	44 (88.0)	43 (97.7)	
Empyema	1 (1.1)	1 (2.0)	–	
Hypoxia	1 (1.1)	1 (2.0)	–	
Pneumomediastinum	1 (1.1)	–	1 (2.3)	
neumothorax	1 (1.1)	1 (2.0)	–	
Respiratory Failure/Death	1 (1.1)	1 (2.0)	–	
Stent Migration	1 (1.1)	1 (2.0)	–	
Other	1 (1.1)	1 (2.0)	–	

Data shown as mean + SD or number (%); *p < 0.05 compared to unsuccessful therapeutic bronchoscopy group

There was no difference in peri-procedural hospital length of stay between the successful and unsuccessful groups (3 ± 4 days vs. 4 ± 6 days, *p* = 0.32, Table 1). Patients in the successful group had improved chest radiographic imaging after the intervention compared to the unsuccessful group (78% vs. 31%, *p* < 0.001, Table 3), but there was no difference in the peri-procedural antibiotic use (16% vs. 18%, *p* = 0.77, Table 1). The patients in the successful vs. unsuccessful groups received adjuvant chemotherapy (42% vs. 50%, *p* = 0.71, Table 1) and radiation (38% vs. 50%, *p* = 0.40, Table 1) as directed by the oncology providers but there was no significant difference.

We used stepwise selection to fit the logistic regression model from the pool of clinically interesting variables which were associated with success of therapeutic bronchoscopy (Table 4). Radiographic distal airway patency was associated with high odds of success, controlling for other variables (OR 11.97, 95% CI:2.56–55.69, *p* = 0.002). Increased time from radiographic finding of CAO to therapeutic bronchoscopy was associated with lower odds of success in the multivariate analysis (OR 0.96, 95% CI: 0.92–1.00, *p* = 0.048).

**Table 4:** Variables associated with Success of Therapeutic Bronchoscopy: Unadjusted and Adjusted Logistic Regression Analysis

Characteristic	Unadjusted Odds Ratio	*p* value	Adjusted Odds Ratioª	*p* value
Smoking Status				
Nonsmoker vs Current Smoker	5.36 (CI 1.45–19.74)	0.01*	3.77 (CI 0.64, 21.96)	0.140
Nonsmoker vs Former Smoker	2.05 (CI 0.69–6.14)	0.19	1.13 (CI 0.27, 4.61)	0.860
Radiographic Distal Airway Patency	15.11 (CI 4.98–45.83)	< 0.001*	11.95 (CI 2.56–55.69)	0.002*
Bronchoscopic Distal Airway Patency	10.77 (CI 3.63–31.95)	< 0.001*	3.55 (CI 0.89–14.17)	0.073
Time to Intervention from Radiographic CAO (days)	0.99 (CI 0.96–1.01)	0.71	0.96 (0.92–1.00)	0.048*

*CI* Confidence interval. *Indicates statistically significant findings. ªAdjusted for age, gender, BMI, smoking status, radiographic distal airway patency, bronchoscopic distal airway patency, radiologic collapse status, tumor histology, mass location, obstruction type, severity of obstruction, time from radiographic finding to intervention, and previous chemotherapy and radiation

Over the study period, the majority of therapeutic bronchoscopies were completed without significant complications (92.6%). A total of 7 complications were observed, including empyema, hypoxic respiratory failure, pneumomediastinum, pneumothorax, and stent migration. One patient (1.1%) developed respiratory failure resulting in death (Table 3).

**Fig. 1:**
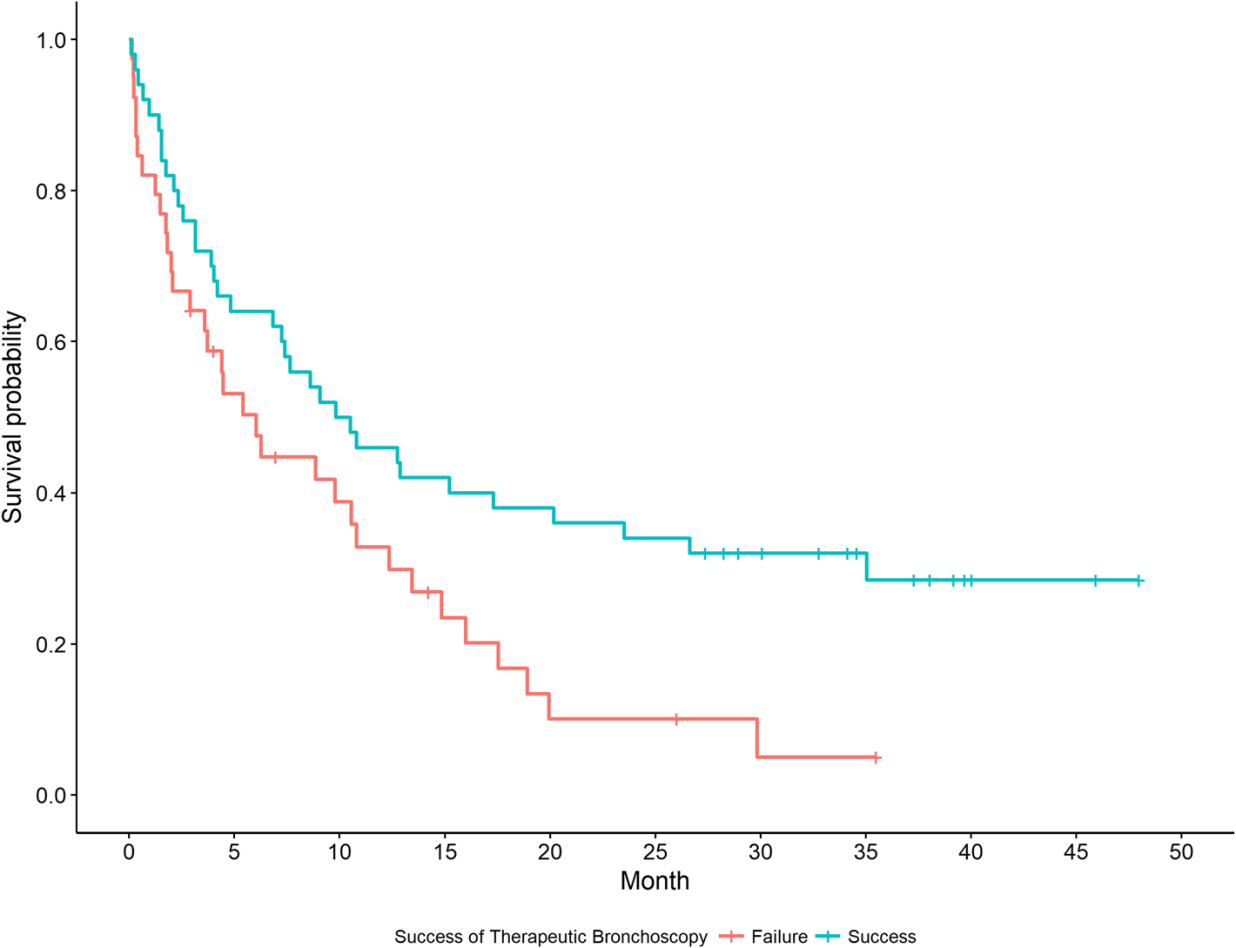
Kaplan Meier Curve demonstrating survival among successful vs. unsuccessful therapeutic bronchoscopy groups. Hazard ratio = 0.55, 95% CI: 0.34–0.90, *p* = 0.017

Of the patients observed in our data set, we gathered follow up data from a total of 89 therapeutic bronchoscopies (39 in the unsuccessful group and 50 in the successful group). Five patients were lost to follow up. A higher percentage of patient deaths were observed in the unsuccessful therapeutic bronchoscopy group compared to the successful group (*n* = 33, 84.6% vs. *n* = 35, 70.0%). Median overall survival was 7.4 months in the cohort, with survival significantly longer in the successful group (10.2 months, 95% CI: 4.8–20.2) compared to the unsuccessful group (6.1 months, 95% CI: 2.1–10.8, log rank *p* = 0.015, Fig. 1).

## Discussion

When successful, therapeutic bronchoscopy for the relief of malignant CAO can ameliorate debilitating symptoms and positively impact quality of life [3, 8, 11, 16, 17]; however, as this is generally performed in high risk patients [18, 19], identifying clinical predictors associated with successful therapeutic bronchoscopy is of utmost importance in procedural planning [2, 18].

This study evaluates clinical and radiographic factors associated with technical success of therapeutic bronchoscopy to relieve malignant CAO. We found the following factors are associated with higher odds of therapeutic success: 1) CT imaging demonstrating a patent airway distal to the CAO prior to intervention, 2) Patent airway visualized distal to the CAO during bronchoscopy, 3) Non- or former-smoking status prior to therapeutic bronchoscopy and 4) Reduced time from the radiographic diagnosis of CAO to therapeutic intervention.

Successful therapeutic bronchoscopy for the relief of CAO, defined as achieving an airway patency > 50% of the lumen, was achieved in 85.4% of cases, consistent with rates of success previously reported within the literature [3, 13, 21]. A multimodality approach to relieve central airway obstruction was commonly utilized. In the successful group, 27/50 (54%) patients had stent placement to establish durable patency. No patient in the unsuccessful group received a stent, as airway patency could not be established and is essential for stent deployment. Also, KTP laser was used for 11 patients in the successful group and 1 patient in the unsuccessful group, primarily for hemostasis (22% vs. 2%, *p* = 0.004). On the other hand, APC was used in 15 patients in the successful group and 24 patients in the unsuccessful group (30% vs. 54%, *p* = 0.01). The association of KTP with success or APC with failure cannot be drawn from this small cohort.

We found that a radiographically patent airway seen distal to the central airway obstruction on CT chest prior to attempted therapeutic intervention, with or without the presence of associated atelectasis, was both more common in the successful group and also associated with significantly greater chances of a technically successful therapeutic bronchoscopy. CT imaging of the chest is widely accepted as an essential component in the evaluation of malignant CAO for not only defining the location, extent and type of airway obstruction but also for procedural planning [1, 2, 6, 22]. Interestingly, in one retrospective study of patients undergoing therapeutic bronchoscopy for CAO, Harris et al. found that airway obstruction on CT chest was often overlooked, omitted from 31% of radiology reports, and resulted in a significant delay in time to bronchoscopy (21 versus 10 days) for patients where the CAO was not reported [23]. Our study also highlights the importance of early radiographic identification of malignant CAO and prompt therapeutic intervention. We found that, when adjusted for other significant variables, each day of delay to therapeutic bronchoscopy from the radiographic CAO diagnosis decreased the probability of successful therapeutic intervention by 4%. Ong et al. also reported that earlier therapeutic bronchoscopy in malignant CAO led to improved quality adjusted survival [17]. This underscores the importance to intervene with therapeutic bronchoscopy in appropriately selected patients as soon as possible.

In addition to radiographic findings, a patent airway distal to the CAO visualized during therapeutic bronchoscopy was independently associated with significantly increased odds of therapeutic success. In a similar retrospective study evaluating the prognostic factors for survival in 2014 patients undergoing therapeutic bronchoscopy, Guibert et al. found that most technical failures (24/26) were related to an inability to traverse or identify a patent airway distal to the malignant CAO during bronchoscopy [21]. Taken together, our findings suggest that careful pre-procedural planning is imperative in considering therapeutic bronchoscopy for malignant CAO, given that a distal patent airway visualized either on CT chest or bronchoscopically can improve the odds of technical success in relieving malignant CAO.

To the best of our knowledge, this is the first study to describe that smoking status was significantly associated with odds of technical success of therapeutic bronchoscopy. Compared to current smokers, never smokers had five times the odds of successful relief of the malignant CAO during therapeutic bronchoscopy. In addition, no significant difference was found between former smokers and never smokers. While technical success does not necessarily convey a meaningful symptomatic improvement [11, 13], our findings are concordant with those of Ost et al. who reported that among patients undergoing therapeutic bronchoscopy for malignant CAO, smokers were less likely to have improvements in dyspnea after their procedure compared to never-smokers [13]. Additionally, Ernst et al. previously found that current tobacco use was independently associated with significantly increased chance of complications during therapeutic bronchoscopy for malignant CAO [19]. Certainly, current smoking alone should not be considered as prohibitive for therapeutic bronchoscopy; however, our study adds to the growing body of literature suggesting that smoking should be considered as a potential predictor for lower technical success, less symptomatic benefit and increased risk of complications. Therefore, patients should be encouraged to quit smoking to optimize outcomes of therapeutic bronchoscopy.

In addition to a correlation with symptom relief, numerous studies have found that successful therapeutic bronchoscopy is associated with improved survival in both malignant and non-malignant CAO [3, 9, 16, 21]. Concordantly, we found a lower rate of death and significantly longer survival in the group undergoing successful relief of malignant CAO, with a hazard ratio of death in the successful group approximately half of that in the unsuccessful group. Similar outcomes were reported by Chhajed et al. in their study of 52 patients undergoing therapeutic bronchoscopy for malignant CAO. They noted that patients with NSCLC related CAO who underwent successful therapeutic bronchoscopy had a median survival of about 8 months, which was not significantly different from patients with advanced NSCLC without CAO [12]. The median survival in their study was comparable to our findings. Successful relief of CAO may prevent or delay morbid complications such as post-obstructive pneumonia, sepsis, respiratory failure and asphyxiation, explaining the potential survival benefit [9, 16], making early identification of predictors of successful therapeutic bronchoscopy critical.

Though our study was performed at a large-volume center for interventional bronchoscopy, it has several limitations including a retrospective design. We did not collect objective data on changes in patient symptoms or quality of life with the interventions; however, this information was previously reported on a subset of our patients [3]. In addition, radiographic and visual assessment of the severity of CAO was used, which can be imprecise but is the standard of care and used routinely in other studies [3, 23, 24]. Although bronchoscopic debridement techniques and ablative modalities have not changed significantly over last 10 years [14], oncologic therapies have improved and can impact survival over the study period. However, we cannot conclusively determine this in our small cohort and a large, multicenter study could evaluate this in the future. Finally, it is possible that some of the significant predictors for decreased success of therapeutic bronchoscopy including higher prevalence of smoking and severity of airway obstruction may convey important physiologic differences between successful and unsuccessful patient groups and should be investigated in the future. Further research is also needed to assess if our reported clinical and radiographic predictors of success in therapeutic bronchoscopy for malignant CAO hold true in larger prospective studies and lead to decreased procedural complications in patients who are unlikely to benefit from the intervention.

## Conclusions

Predictors associated with success of therapeutic bronchoscopy for relief of malignant CAO include distal airway patency on pre-procedural CT imaging and flexible bronchoscopy. Better odds of achieving airway patency is seen in non- or former-smokers, and with decreased time from radiographic finding of CAO to bronchoscopic intervention.

## Data Availability

All datasets used and/or analyzed during the current study are available from the corresponding author on reasonable request.

## Abbreviations

APC: Argon plasma coagulation
CAO: Central airway obstruction
KTP: Potassium titanyl phosphate
NSCLC: Non-small cell lung cancer
SCLC: Small cell lung cancer

## References

[1] Ernst A, Feller-Kopman D, Becker HD, Mehta AC (2004). Central airway obstruction. Am J Respir Crit Care Med.

[2] Murgu SD, Egressy K, Laxmanan B, Doblare G, Ortiz-Comino R, Hogarth DK (2016). Central airway obstruction: benign strictures, Tracheobronchomalacia, and malignancy-related obstruction. Chest.

[3] Mahmood K, Wahidi MM, Thomas S, Argento AC, Ninan NA, Smathers EC, Shofer SL (2015). Therapeutic bronchoscopy improves spirometry, quality of life, and survival in central airway obstruction. Respiration.

[4] Shin B, Chang B, Kim H, Jeong BH (2018). Interventional bronchoscopy in malignant central airway obstruction by extra-pulmonary malignancy. BMC Pulm Med.

[5] Semaan R, Yarmus L (2015). Rigid bronchoscopy and silicone stents in the management of central airway obstruction. J Thorac Dis.

[6] Mudambi L, Miller R, Eapen GA (2017). Malignant central airway obstruction. J Thorac Dis.

[7] Neyman K, Sundset A, Espinoza A, Kongerud J, Fosse E (2011). Survival and complications after interventional bronchoscopy in malignant central airway obstruction: a single-center experience. J Bronchology Interv Pulmonol.

[8] Oviatt PL, Stather DR, Michaud G, Maceachern P, Tremblay A (2011). Exercise capacity, lung function, and quality of life after interventional bronchoscopy. J Thorac Oncol.

[9] Razi SS, Lebovics RS, Schwartz G, Sancheti M, Belsley S, Connery CP, Bhora FY (2010). Timely airway stenting improves survival in patients with malignant central airway obstruction. Ann Thorac Surg.

[10] Haas AR (2007). Recent advances in the palliative management of respiratory symptoms in advanced-stage oncology patients. Am J Hosp Palliat Care.

[11] Amjadi K, Voduc N, Cruysberghs Y, Lemmens R, Fergusson DA, Doucette S, Noppen M (2008). Impact of interventional bronchoscopy on quality of life in malignant airway obstruction. Respiration.

[12] Chhajed PN, Baty F, Pless M, Somandin S, Tamm M, Brutsche MH (2006). Outcome of treated advanced non-small cell lung cancer with and without central airway obstruction. Chest.

[13] Ost DE, Ernst A, Grosu HB, Lei X, Diaz-Mendoza J, Slade M, Gildea TR, Machuzak MS, Jimenez CA, Toth J (2015). Therapeutic bronchoscopy for malignant central airway obstruction: success rates and impact on dyspnea and quality of life. Chest.

[14] Mahmood K, Wahidi MM (2014). Ablative therapies for central airway obstruction. Semin Respir Crit Care Med.

[15] Gompelmann D, Eberhardt R, Herth FJ (2011). Advanced malignant lung disease: what the specialist can offer. Respiration.

[16] Stratakos G, Gerovasili V, Dimitropoulos C, Giozos I, Filippidis FT, Gennimata S, Zarogoulidis P, Zissimopoulos A, Pataka A, Koufos N (2016). Survival and quality of life benefit after endoscopic Management of Malignant Central Airway Obstruction. J Cancer.

[17] Ong P, Grosu HB, Debiane L, Casal RF, Eapen GA, Jimenez CA, Noor L, Ost DE. Long-term quality-adjusted survival following therapeutic bronchoscopy for malignant central airway obstruction. Thorax. 2019;74(2):141–156. 10.1136/thoraxjnl-2018-211521 30254139

[18] Ost DE, Ernst A, Grosu HB, Lei X, Diaz-Mendoza J, Slade M, Gildea TR, Machuzak M, Jimenez CA, Toth J (2015). Complications following therapeutic bronchoscopy for malignant central airway obstruction: results of the AQuIRE registry. Chest.

[19] Ernst A, Simoff M, Ost D, Goldman Y, Herth FJF (2008). Prospective risk-adjusted morbidity and mortality outcome analysis after therapeutic bronchoscopic procedures: results of a multi-institutional outcomes database. Chest.

[20] Dalar L, Ozdemir C, Abul Y, Karasulu L, Sokucu SN, Akbas A, Altn S (2016). Therapeutic bronchoscopic interventions for malignant airway obstruction: a retrospective study from experience on 547 patients. Medicine (Baltimore).

[21] Guibert N, Mazieres J, Lepage B, Plat G, Didier A, Hermant C (2014). Prognostic factors associated with interventional bronchoscopy in lung cancer. Ann Thorac Surg.

[22] Shiau M, Harkin TJ, Naidich DP (2015). Imaging of the central airways with bronchoscopic correlation: pictorial essay. Clin Chest Med.

[23] Harris K, Alraiyes AH, Attwood K, Modi K, Dhillon SS (2016). Reporting of central airway obstruction on radiology reports and impact on bronchoscopic airway interventions and patient outcomes. Ther Adv Respir Dis.

[24] Egressy KV, Murgu SD (2015). Current approaches to assessing the degree of airway narrowing in central airway obstruction. Ann Am Thorac Soc.

